# Supplementary material for: Improved In Vivo Anti-Tumor and Anti-Metastatic Effect of GnRH-III-Daunorubicin Analogs on Colorectal and Breast Carcinoma Bearing Mice
Source: Int J Mol Sci. 2019 Sep 25;20(19):4763. doi: 10.3390/ijms20194763 (PMC6801585; doi:10.3390/ijms20194763)
Supplement: Supplementary file 1 [file ijms-20-04763-s001.pdf]

International Journal of *Molecular Sciences*

MDPI

Supplementary Part

Improved In Vivo Anti-Tumor and Anti-Metastatic Effect of GnRH-III-Daunorubicin Analogs on Colorectal and Breast Carcinoma Bearing Mice.

Ivan Ranđelović [1], Sabine Schuster [2,3], Bence Kapuvári [4], Gianluca Fossati [5], Christian Steinkühler [5], Gábor Mező [2,3] and József Tóvári [1,*]

1 Department of Experimental Pharmacology, National Institute of Oncology, 1122 Budapest, Hungary; ivan.randel@gmail.com (I.R.)
2 Faculty of Science, Institute of Chemistry, Eötvös Loránd University, 1117 Budapest Hungary, sabine.schuster83@gmail.com (S.S.); gmezo@caesar.elte.hu (G.M.)
3 MTA-ELTE Research Group of Peptide Chemistry, Hungarian Academy of Sciences, Eötvös Loránd University, 1117 Budapest, Hungary; sabine.schuster83@gmail.com (S.S.); gmezo@caesar.elte.hu (G.M.)
4 Department of Biochemistry, National Institute of Oncology, 1122 Budapest, Hungary; zeuglodon@freemail.hu (B.K.)
5 Preclinical R&D, Italfarmaco SpA, 20092 Cinisello Balsamo (Milan), Italy; g.fossati@italfarmaco.com (G.F.); c.steinkuhler@italfarmaco.com (C.S.)
* Correspondence: jtovari@yahoo.com (J.T.); Tel.: +36-1-224-8778

Received: date; Accepted: date; Published: 27 September 2019

Table of Content

Stability of GnRH-III-Dau conjugates in mice plasma 2

GnRH-R mRNA, absolute protein and cell surface expression level compared to MRC-5 and cellular uptake rates of conjugates 1 and 2 3

Spleen weight in 4T1 tumor bearing mice 4

Stability of GnRH-III-Dau conjugates in mice plasma

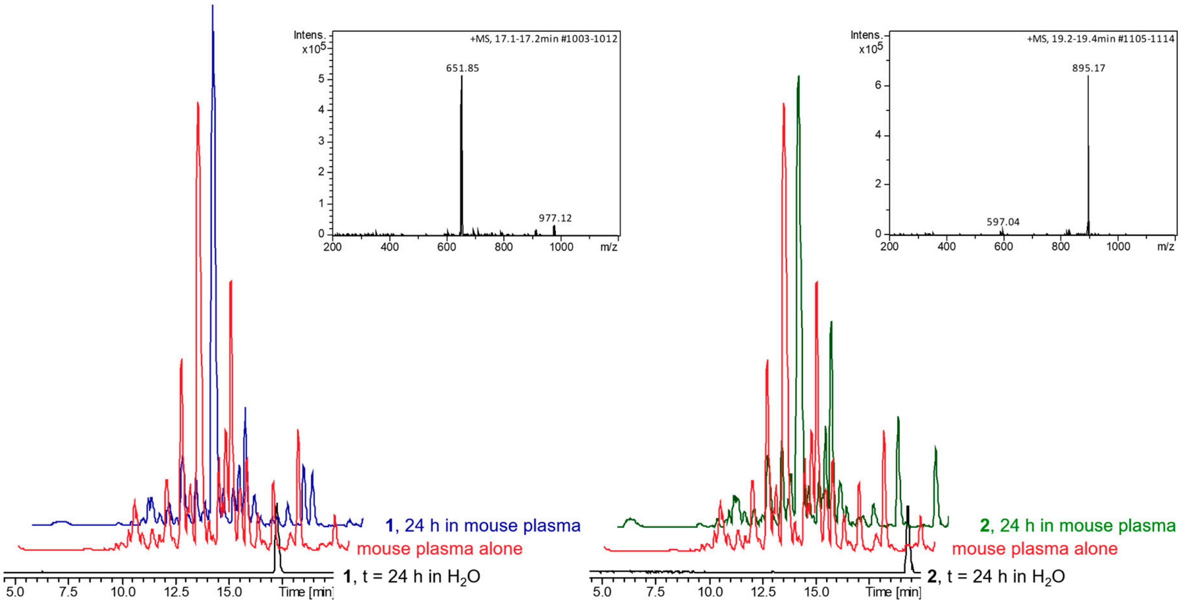

Figure S1. Stability of the GnRH-III-Dau conjugates **1** and **2** in mouse plasma. LC-MS chromatogram of mouse plasma and the conjugates after 24 hours incubation at 37 °C in ddH2O or in human plasma plus the corresponding MS compound spectra.

*GnRH-R mRNA, absolute protein and cell surface expression level compared to MRC-5 and cellular uptake rates of conjugates **1** and **2***

Table S1. GnRH-R mRNA, absolute protein and cell surface expression level compared to MRC-5. Cellular uptake of conjugates **1** and **2** (40 μM), after 6 h treatment of the cell lines. Values from 2 independent experiments are shown.

	MRC-5	MDA-MB-231	HT-29	A2780	PANC-1	U87MG	4T1
	1.0	5.1	3.3	1.6	1.4	0.5	n.d.
mRNA	1.0	9.5	5.9	5.3	2.2	1.1	n.d.
	1.0	1.5	2.7	5.8	0.9	4.5	4.3
Protein	1.0	1.0	0.6	2.0	0.5	2.6	2.5
	1.0	6.3	2.2	6.5	4.7	3.1	4.2
Cell surface	1.0	5.0	1.8	4.7	3.4	4.3	3.4
	8.1	12.0	12.4	12.2	5.8	12.9	13.0
Uptake Conjugate 1	8.3	18.2	22.4	22.3	6.4	14.8	21.1
	14.9	19.9	21.0	26.3	15.4	22.3	26.8
Uptake Conjugate 2	15.3	34.0*	38.3*	42.8**	17.9*	26.8*	38.8**

n.d. = not determined. [1] Relative GnRH-R mRNA expression normalized to β-actin and GAPDH. [2] Relative GnRH-R protein expression normalized to β-actin. [3] Relative GnRH-R Geo MFI. [4] Relative Geo MFI normalized to auto fluorescence. Statistical analysis for uptake studies of conjugates **1** and **2** was performed by 2way ANOVA test. * and ** mean significant at $p<0.05$ and $p<0.01$, respectively.

Spleen weight in 4T1 tumor bearing mice

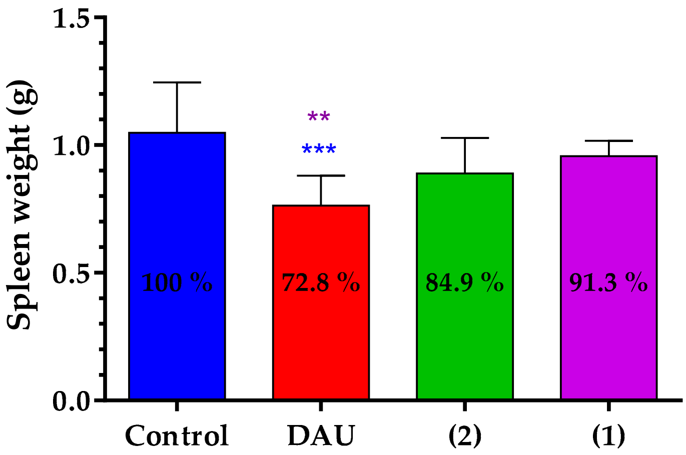

Figure S2. Spleen weight in 4T1 tumor bearing mice (grams, average ± SD) after termination of experiment, 28 days subsequent to cells inoculation, 7 animals per group, statistical analysis was performed by Mann-Whitney test. ** and *** mean significant at $p<0.01$, and $p<0.001$, respectively. * blue and purple mean significant difference compared to control and 1 groups respectively.

© 2019 by the authors. Licensee MDPI, Basel, Switzerland. This article is an open access article distributed under the terms and conditions of the Creative Commons Attribution (CC BY) license (http://creativecommons.org/licenses/by/4.0/).
